# Use of outpatient mental healthcare services and upper-secondary school completion in young women with migrant background – A population-based study

**DOI:** 10.1016/j.ssmph.2020.100631

**Published:** 2020-07-15

**Authors:** Kamila Angelika Hynek, Melanie Straiton, Lars Johan Hauge, Karina Corbett, Dawit Shawel Abebe

**Affiliations:** a Norwegian Institute of Public Health, Postboks 222 Skøyen, 0213, Oslo, Norway; b Oslo Metropolitan University, Pilestredet 32, 0166, Oslo, Norway

**Keywords:** Outpatient mental healthcare services, Upper-secondary school completion, Migrants, Young women

## Abstract

Mental disorders typically develop during adolescence, with young women being particularly at risk. Mental disorders during this period can negatively affect both current and future life prospects such as school completion. Migrants are at increased risk of developing mental disorders as a result of their experiences prior to, during and after migration. Additionally, they are less likely to complete upper-secondary school when compared to the majority population. Thus, being a young migrant woman with a mental disorder may have adverse consequences for school completion, which in turn can affect socioeconomic status later in life. In this study, we aimed to investigate the association between mental disorders, defined as having used outpatient mental healthcare services (OPMH), and completion of upper-secondary school among young women living in Norway, using national registry data. Additionally, we examined differences in probability of school completion between Norwegian majority, migrants and migrant descendants between those who used and did not use OPMH. The sample consisted of women born between 1990 and 1993 (N = 122,777). We conducted hierarchical, multivariable logistic regression analysis. In unadjusted analysis, we found that young women who used OPMH services had lower odds of school completion than those who did not, even after adjustment for migrant background and parental education. However, by calculating predictive margins, we found that descendant women, who had used OPMH services, had significantly higher probability of completing upper-secondary education than Norwegian majority women who had used services. None of the four migrant groups differed significantly from majority women. Use of OPMH services, had most adverse effect on majority, migrants from Nordic and Western countries and descendants, when compared to non-users. Future interventions should aim to increase school completion among young women with mental disorders.

## Highlights

• This is a cohort study of young women in Norway, using national registry data.
• Mental health service use reduces the odds of upper-secondary school completion.
• Migrant women using services do not have lower rates of completion than majority.
• School noncompletion have lasting socioeconomic consequences.

## 1 Introduction

Mental disorders are a public health challenge. Globally about 16% were affected by a mental or addictive disorder in 2016 (Rehm & Shield, 2019). In Norway, almost a quarter of young women aged 16–24 years report significant mental problems (Nes & Clench-Aas, 2011; Norhealth, n.d.), making them the most vulnerable group. Several mental disorders often have an onset during the teenage years (Jones, 2013), due to biological, cognitive and emotional changes during adolescence (Lee et al., 2014). The research focus on mental disorders in young women is important, not only due to the impact on current life but also in the long term. Poor mental health negatively influences other health outcomes and increases the risk of school noncompletion (Bowman, McKinstry, & McGorry, 2017). In turn, this can further result in lower salary, poorer workforce attachment and increased dependence on welfare services in adulthood (Ngui, Khasakhala, Ndetei, & Roberts, 2010; Trautmann, Rehm, & Wittchen, 2016).

As well as young women, migrants are at increased risk of mental disorders as a result of migration-related experiences (Delara, 2016). Worldwide, the number of international migrants is rapidly growing (United Nations, 2017) and Norway is no exception. The migrant population in Norway has increased significantly over the past decades (Sandnes, 2017). The combination of being young, a woman and a migrant may therefore have adverse effects on mental health. Cultural differences regarding gender roles, lack of social support and discrimination may not only increase the risk of mental disorders (Abebe, Lien, & Hjelde, 2014; Llácer, Zunzunegui, Del Amo, Mazarrasa, & Bolůmar, 2007), but may also have a negative impact on socioeconomic outcomes, such as school completion. In Norway, young migrant women have lower upper-secondary school completion rates compared to both majority and descendant women (Steinkellner, 2017). However, we lack information on the impact of mental disorders on school completion among this group. In this study, we aim to investigate whether there is an association between mental disorders, as measured by the use of outpatient mental healthcare services (OPMH), and upper-secondary school completion.

### 1.1 Previous research

Research on the association between mental disorders and school completion or other school-related outcomes is not new (Kessler, Foster, Saunders, & Stang, 1995). Two systematic reviews summarizing research on this topic concluded that mental disorders were negatively associated with school completion and educational attainment and positively associated with early school leaving (Esch et al., 2014; Melkevik, Nilsen, Evensen, Reneflot, & Mykletun, 2016). However, authors point to the potential mediating effect of socioeconomic background and family support on these associations (Esch et al., 2014). Despite focusing on different types and severity of mental disorders, the majority of studies indicate that mental disorders have a negative impact on educational attainment such as school completion, college enrolment and academic performance (Brännlund, Strandh, & Nilsson, 2017; Breslau, Lane, Sampson, & Kessler, 2008; Leach & Butterworth, 2012; McLeod, Uemura, & Rohrman, 2012; Mojtabai et al., 2015; Myer et al., 2009; Needham, 2009; Van der Ende, Verhulst, & Tiemeier, 2016). It is important to stress that few of the above studies stratified findings by gender and most relied on self-reported data. Studies that did investigate gender differences found poor mental health to have a stronger negative effect on school completion among young women than young men (Brännlund et al., 2017; Fletcher, 2008; Needham, 2009).

The above studies can be placed under the *social selection perspective*, which assumes that mental disorders have a detrimental effect on later life achievements and maintenance of social status (Mossakowski, 2014). Previous research pinpointed that the presence of social selection is strongest for disorders that are serious or socially disruptive, such as schizophrenia, antisocial personality disorder and conduct disorders (McLeod & Kaiser, 2004; Mossakowski, 2008). The relationship between mental disorder and school completion may alternatively be *confounded* by other factors associated with both mental disorder and school completion. Lynch and von Hippel (2016) investigated these explanations for self-rated health and education. They concluded that good self-rated health in adolescents predicted higher educational attainment (i.e., the social selection mechanism) but that family background and college aspirations partly confounded this association. Similarly, the association between mental disorder and educational attainment may be confounded by family background variables. Family socioeconomic status (SES), such as parental educational attainment for instance, is associated with both risk of mental disorder in (Reiss, 2013), and educational attainment of children (Davis-Kean, 2005). Parental country of birth has also been used as an indicator for family SES and is a potential confounder in the association between mental disorder and school completion (Brännlund et al., 2017). Studies investigating the association between use of OPMH services, or mental disorders, and school completion should control for confounding factors in order to measure the possible direct effect of mental disorders on school completion.

Norwegian research on the association between mental disorders and school completion mainly relies on self-reported mental disorders. Self-reported mental disorders are associated with non-completion of upper-secondary school (Sagatun, Heyerdahl, Wentzel-Larsen, & Lien, 2014). Furthermore, adolescent mental disorders reduce years of schooling, and the probability of upper-secondary school completion and college enrolment (Evensen, Lyngstad, Melkevik, & Mykletun, 2016). These studies have not focused on young women in particular nor considered differences between majority populations and migrants. To the best of our knowledge, there is a lack of research looking at the relationship between mental disorders, treated at outpatient level, and school completion among women with migrant background. Our study will contribute to filling the gap in the existing literature by looking at OPMH service use and upper-secondary school completion among young majority, migrant and descendant women.

### 1.2 Migrant women in Norway

Norway, like several other European countries, has experienced an increase in the number of migrants during recent decades. Between 1970 and 2017, the percentage of migrants rose from about one percent to more than 14% of the total Norwegian population (Dzamarija, 2017). Together with their descendants, migrants made up almost 18% of the population in 2019 (Statistics Norway, 2019b). Depending on country of origin and reason for migration, migrants may have a different risk of mental disorder and use of mental healthcare services. Migrants, particularly those from low- and middle-income countries, may be at greater risk of mental health problems compared to the majority population (Abebe et al., 2014). There are also differences in use of primary healthcare services (PHC) for mental health problems where, for instance, refugee women have higher use than non-refugee women (Straiton, Reneflot, & Diaz, 2017). Use of PHC also varies by country of origin, though migrant women in general have lower usage compared to their Norwegian counterparts (Straiton, Reneflot, & Diaz, 2014). Use of OPMH services is also lower for both migrant and descendant women compared to their Norwegian counterparts, although again there is considerable variation by country of origin (Straiton, Corbett, Hollander, & Hauge, 2019).

Regarding school completion, young migrant women are more likely to drop out of upper-secondary school and less likely to complete school within the normative time frame of five years, compared to descendants and the Norwegian majority (Statistics Norway, 2019a). Among descendant women, the dropout percentage is lower compared to majority women, though, a lower percentage complete upper-secondary school within the normative time frame (Statistics Norway, 2019a). Among those who do complete upper-secondary school education, a higher percentage of descendant women enrol in higher education compared to majority women (Steinkellner, 2017).

### 1.3 Current study

Previous research on the association between mental disorders and school completion is mainly based on survey studies, which are known for being prone to selection bias due to low response rates among migrants and minority populations (Llácer et al., 2007). Furthermore, none of the above studies investigated whether the effect of OPMH service use on school completion varies between majority, migrant or descendant young women. In the current study, we use register data with national level coverage to look at upper-secondary school completion among young women who have used OPMH services, as an indicator of mental disorders, and those who have not. We also control for migrant background and parental education to look at whether these factors confound the association.

Additionally, we examine whether the probability of completing upper-secondary school among those using and not using OPMH services, varies between young majority, migrant and descendant women. Based on the available evidence on migrant women's lower use of healthcare services, despite increased risk of mental disorders, and lower rates of school completion compared to both descendants and Norwegian majority, we hypothesize that young migrant women will have lower probability of completing upper-secondary school when using OPMH services than majority and descendant women. We also expect probabilities to vary among migrants, depending on their region of origin and when compared to non-users within the same group.

## 2 Methods

### 2.1 Study design and data sources

In this cohort study of young women in Norway, we used longitudinal population-based data from several Norwegian registries, namely the Central Population Registry, the National Database for the Reimbursement of Health Expenses (KUHR) and the National Education Database. The Central Population Registry provided demographic information such as year of birth, sex and country of origin. Additionally, it contains information on time of immigration, emigration or death, and thus, whether an individual resided in Norway during respective years of study. This was used to define the eligible study sample. The KUHR database contains data on healthcare professionals’ reimbursement claims of fee-for-service in Norway, such as claims from OPMH services as used in this study. Each contact with healthcare providers is registered with a date and type of contact. OPMH services are located throughout the country and available for all Norwegian residents. After referral from a general practitioner (GP) or psychologist, individuals with acute problems or those with a need for long-term follow-up care can receive help. We use information on contact with OPMH services for years 2006–2011. From the National Education Database, we used information on the highest obtained education level and any initiated education for years 2011–2014 in order to identify those who did or did not complete upper-secondary education. Additionally, we extracted information on parental education when the individual was aged 16.

A unique personal identification number (PIN) is assigned to all Norwegian citizens at birth, as well as all individuals registered as residents in Norway for at least six months. We used a pseudonymised version of a PIN to combine variables across the registries necessary to examine the objectives of interest. We also used family number to extract the information on parental education.

### 2.2 Study sample

The study population consisted of 149,271 young women born between 1990 and 1993. We excluded individuals who died (N = 993) or emigrated permanently (N = 5,319) at the age of 18 or earlier, as they did not meet inclusion criterion. We also excluded those who immigrated to Norway at the age of 18 or later (N = 17,043). Furthermore, we included only young women who started upper-secondary education and resided in Norway during the exposure period (excluded N = 2,879). We excluded women from South America due to a small number of individuals in this group (N = 260). The eligible study sample for the aims of this study constituted of 122,777 young women. We followed each of them for five years, between the age of 16 and 21.

### 2.3 Outcome variable

*Upper-secondary education* was used as outcome variable and was defined as completion of upper-secondary education by the age of 21 and treated as a binary variable in the analyses. In Norway, all youths between 16 and 19 have the right to start upper-secondary education after completing compulsory education, with the majority of students starting upper-secondary education the year they turn 16. The Norwegian school system is largely public and free of charge. Compulsory education lasts for 10 years, when aged 6–16 years. All children who have stayed in Norway for at least three months have the right and duty to attend compulsory education until summer the year they turn 16. After completing compulsory education, all students have the right to continue into upper-secondary education, which is free of charge when public. Upper-secondary education is divided into general and vocational tracks. The general track lasts for three years and, after passing all required exams, is a qualification for tertiary education. The vocational track has three pathways lasting for three or four years. Two of them give qualifications for practical occupations, without qualification for tertiary education, while the third path leads to a tertiary education qualification, if specific required courses are taken. Due to variations in the length of study programs, the normative time frame for upper-secondary school completion is set to five years i.e. by the age of 21 (Statistics Norway, 2019a).

### 2.4 Exposure variable

**Fig. 1:**
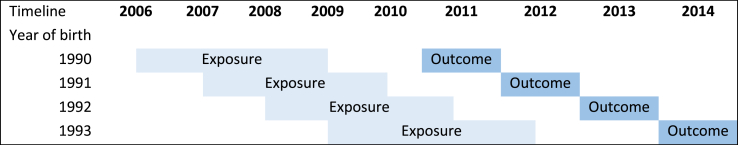
Timeline for the study, with point of measurement of exposure, OPMH service use, and outcome, upper-secondary school completion, for each of age cohorts.

*OPMH service use* was utilized as our exposure variable and an indicator of mental disorders. We employed a dichotomous indicator (yes/no) to identify individuals with at least one contact with OPMH services during late adolescence (aged between 16 and 19), with a start point on 1^st^ of July and end point on 30^th^ of June, three years later, as shown in Fig. 1.

### 2.5 Covariates

#### 2.5.1 Migrant background

We divided our sample into three main groups: migrant (born abroad to two foreign-born parents), descendant (Norwegian-born to two foreign-born parents) and majority women (Norwegian-born with at least one Norwegian-born parent, and foreign-born with at least one Norwegian-born parent). We further divided migrants into four categories as 1) Nordics and Western countries (including Nordic countries, Western Europe, USA, Canada, Australia and New Zealand), 2) Eastern Europe, 3) Africa and 4) Asia.

#### 2.5.2 Parental education

Parental education refers to the mother or father's highest attained level of education by the time the individual was aged 16. This variable had three possible values: upper-secondary school or lower, tertiary education, and unknown parental education.

### 2.6 Analyses

By using chi-square tests and post-hoc Bonferroni tests for analysis of variance, we compared the characteristics of descendants and each migrant group to majority women as a reference group. To investigate the association between use of OPMH services and upper-secondary school completion among all young women we used a hierarchical, multivariable logistic regression model. First, we applied univariate logistic regression model, and thereafter adjusted for migrant background and parental education to control for confounding, in model 2 and 3 accordingly. Furthermore, in model 4, we tested for statistical interactions between use of OPMH services and migrant background in order to examine whether the effect of use of OPMH services on school completion varies with migrant background. We compared young women who did and did not use OPMH services with different migrant backgrounds by using predictive margins. Results are presented as predicted probabilities (Pr) with 95% confidence intervals (95% CI). This eased the interpretation of results as predictive margins compare the predicted probabilities of school completion for all groups (Williams, 2012). The analyses were conducted with inclusion of individuals with unknown parental education, as treating this category as missing would make the groups of migrants smaller, resulting in more imprecise and wider confidence intervals. An additional analysis with complete cases did not change the direction of the results or show visual changes in the main outcome. Hence the risk of bias due to missingness seems to be limited. Stata 15.0 was used to execute the analyses.

### 2.7 Ethics

This project has been granted approval by the Regional Committee for Medical and Health Research Ethics, South East Norway (REK 2014/1970) and all registry owners approved the use of their data.

## 3 Results

Table 1 shows that of the 122,777 young women, 91.6% were classified as Norwegian majority, 5.1% as migrants divided into four groups, and 3.3% as descendants. There were significant differences in use of OPMH services; with a higher percentage of Norwegian majority using services compared to descendants, and migrant women from Eastern Europe, Asia and Africa but not migrant women from Nordic and Western countries (X^2^ = 595.0, df = 5, p < 0.001). We also found significant differences between majority women and all groups of migrants, but not descendants, regarding school completion (X^2^ = 244.3, df = 5, p < 0.001). Parental tertiary education was most common among migrant women from Nordic and Western countries and the Norwegian majority, while parental upper-secondary education or lower was most common among descendants and migrants from Africa. All groups differed significantly from the majority (X^2^ = 2300.0, df = 5, p < 0.001).

**Table 1:** Descriptive statistics of the study population, by migrant category.

	**Total sample (N** = **122,777)**	**Majority**^a^**(N** = **112,424)**	**Descendants (N** = **4,035)**	**Migrants (N** = **6,318)**	Nordics and Western countries (N = 895)	Eastern Europe (N = 1,918)	**Asia (N** = **2,502)**	**Africa (N** = **1,003)**
Percentage of the study population		91.6%	3.3%	5.2%	0.7%	1.6%	2.0%	0.8%
Percentage of migrants		–	–	–	14.2%	30.4%	39.6%	15.9%
OPMH service use [N (%)]								
p-value			<0.001	<0.001	1.000	<0.001	<0.001	0.006
Yes	16,638 (13.6)	15,662 (13.9)	326 (8.1)	650 (10.3)	118 (13.2)	173 (9.0)	258 (10.3)	101 (10.1)
No	106,139 (86.4)	96,762 (86.1)	3,709(91.9)	5,668 (89.71)	777 (86.8)	1,745 (91.0)	2,244 (89.7)	902 (89.9)
Upper-secondary education^b^ [N (%)]								
p-value			1.000	<0.001	<0.001	<0.001	<0.001	<0.001
Yes	92,681 (75.5)	85,845 (76.4)	3,049 (75.6)	3,787 (59.9)	551 (61.6)	1,298 (67.7)	1,444 (57.7)	494 (49.3)
No	30,096 (24.5)	26,579 (23.6)	986 (24.4)	2,531 (40.1)	344 (38.4)	620 (32.3)	1,085 (42.3)	509 (50.7)
Parental education [N (%)]								
p-value			<0.001	<0.001	<0.001	<0.001	<0.001	<0.001
Upper-secondary education or lower	68,161 (55.5)	62,135 (55.3)	2,684 (66.5)	3,342 (52.9)	283 (31.6)	961 (50.1)	1,480 (59.2)	618 (61.6)
Tertiary education	53,497 (43.6)	50,261 (44.7)	1,254 (31.1)	1,982 (31.4)	416 (46.5)	708 (36.9)	676 (27.0)	182 (18.2)
Unknown parental education	1119 (0.9)	28 (0.0)	97 (2.4)	994 (15.7)	196 (21.9)	249 (13.0)	346 (13.8)	203 (20.2)

a Reference group.

b By the age of 21; p-values are based on post hoc Bonferroni test, with Norwegian majority as a reference group.

**Fig. 2:**
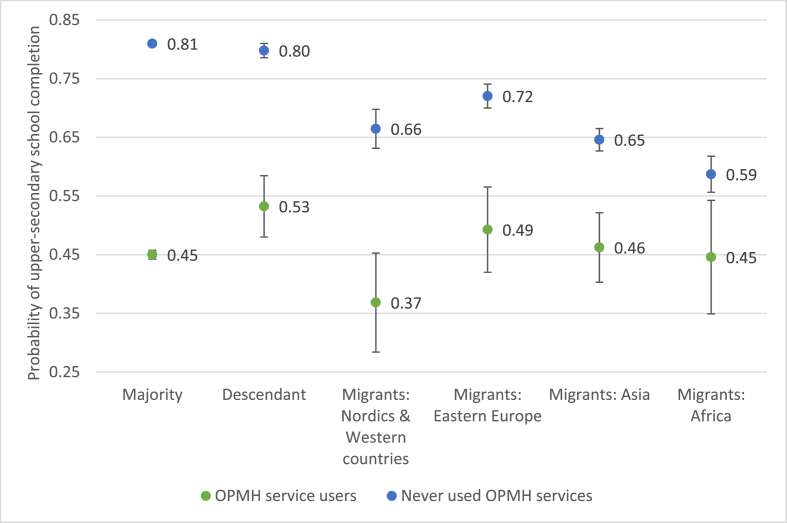
Predicted probabilities with 95% confidence intervals of school completion for women who had contact with OPMH services and those who did not, by migrant background.

In Table 2, we display the results of the hierarchical, multivariable logistic regression analysis conducted to investigate the association between use of OPMH services and upper-secondary school completion. In model 1, the results show the unadjusted analysis, with a clear negative association between use of OPMH services and school completion. Young women who had contact with OPMH services had significantly lower odds of completing upper-secondary school within five years compared to those who did not use OPMH services (OR = 0.20, CI 95% 0.20–0.21). In the adjusted analysis, all control variables were significantly associated with school completion, although they had modest influence on the OR for OPMH services. All four migrant groups and descendants had significantly lower odds of school completion compared to the Norwegian majority (Model 2). However, after controlling for parental education, descendants did not differ significantly from the Norwegian majority, while all migrant groups had significantly lower odds than the Norwegian majority (Model 3). The results showed that having parents with tertiary education increased the odds of school completion compared to having parents with upper-secondary education or lower. In model 4 (results not shown), we conducted interaction analysis between use of OPMH services and migrant background to examine whether the effect of use of OPMH services on school completion differed between groups. The effect of moderation was significant for all groups, indicating the need for additional analyses. To investigate whether the effect of use of OPMH services on upper-secondary school completion varied between the Norwegian majority and the four migrant groups and descendants, we compared predicted probabilities of completing upper-secondary education among the groups by using predictive margins (results are presented in Fig. 2).

**Table 2:** Crude and adjusted odds ratios (OR) with 95% confidence intervals (95% CI) and significance level for the association between use of OPMH services and upper-secondary school completion.

	Model 1		Model 2		Model 3	
	OR [95% CI]	p-value	OR [95% CI]	p-value	OR [95% CI]	p-value
OPMH service use (ref. no)	0.20 [0.20–0.21]	<0.001	0.20 [0.19–0.20]	<0.001	0.19 [0.19–0.20]	<0.001
Migrant background						
Norwegian majority			1.0		1.0	
Descendant			0.84 [0.78–0.91]	<0.001	0.97 [0.90–1.05]	0.499
Migrants:						
Nordics and Western countries			0.46 [0.40–0.53]	<0.001	0.48 [0.41–0.55]	<0.001
Eastern Europe			0.57 [0.51–0.63]	<0.001	0.64 [0.58–0.71]	<0.001
Asia			0.37 [0.34–0.40]	<0.001	0.45 [0.41–0.49]	<0.001
Africa			0.26 [0.23–0.29]	<0.001	0.35 [0.30–0.40]	<0.001
Parental education						
Upper-secondary education or lower					1.0	
Tertiary education					3.00 [2.91–3.09]	<0.001
Unknown parental education					0.68 [0.60–0.78]	<0.001

All models were conducted on the sample of 122,777 individuals. Estimates are odds ratios (OR), with 95% confidence intervals (95% CI) in brackets.

In Fig. 2, results are presented as predicted probabilities with 95% confidence intervals to demonstrate the probability of completing upper-secondary education among young women who did and did not use OPMH services. We present predicted probabilities for the Norwegian majority, descendants and four groups of migrants. Regarding women not using OPMH services, both majority and descendant women had significantly higher probability of school completion when compared to all four groups of migrants. However, when comparing women who used OPMH services, young descendant women (Pr = 0.53, 95% CI 0.48–0.58) had significantly higher probability of completing upper-secondary school within five years when compared to the Norwegian majority (Pr = 0.45, 95% CI 0.44–0-46). Migrants from all four groups did not differ significantly from the Norwegian majority. However, compared to descendant women, migrant women from Nordic and Western countries (Pr = 0.37, 95% CI 0.28–0.45) had significantly lower probability of school completion. Overall, the largest drop in probability of upper-secondary school completion due to use of OPMH services was observed in Norwegian majority, migrant women from Nordic and Western countries and descendant women, with a difference of 36, 29 and 27 percentage points respectively. The probability of school completion for migrant women from Eastern Europe, Asia and Africa was least affected when comparing users and non-users of OPMH services (23, 19 and 14 percentage points respectively).

## 4 Discussion

In this study, we investigated the association between use of OPMH services, an indicator of mental disorders, and upper-secondary school completion among young women in Norway. Furthermore, we aimed to examine whether the probability of completing upper-secondary education when using and not using OPMH services differs for the Norwegian majority, migrants and descendants. We hypothesized that use of OPMH services would have more adverse effect on the probability of completing upper-secondary for migrant women, compared to descendants and the Norwegian majority. We also expected probabilities to vary among migrants, depending on their region of origin and when compared to non-users within the same group.

The results showed that being in contact with OPMH services during adolescence reduced the odds of upper-secondary school completion for all young women in our study sample. The negative association remained after adjustment for migrant background and parental education. By use of predictive margins, we compared the predicted probabilities of school completion when using and not using OPMH services for all groups of migrants, descendants and the majority. Descendants who had used OPMH services appeared to fare significantly better than their Norwegian majority counterparts, but also migrant women from Nordic and Western countries in terms of school completion. Contrary to our expectations, none of the four migrant groups differed significantly from the Norwegian majority when comparing OPMH service users. We also found that within groups, use of OPMH services had the most adverse effects on school completion among majority, migrants from Nordic and Western countries and descendants, when compared to non-users.

Our main findings are in accordance with earlier research on the field, claiming that young women experiencing mental disorders are at greater risk of not completing upper-secondary education compared with their healthy counterparts (e.g. Fletcher, 2008; Needham, 2009). This supports the social selection perspective. The previous studies we refer to relied on self-reported mental health measures collected through surveys. The results are therefore prone to both recall bias and selection effects, since those who choose to participate may differ from those who choose not to. Brännlund et al. (2017), however, based their study on objective data from Swedish registries but with drug prescription as a proxy for mental disorders and also found a negative association between mental disorders and educational achievement. Yet, none of these studies considered differences between adolescents or young women with different migrant background. Previously, it has been pointed out that surveys often suffer from bias due to lower response rates among minority populations, mainly due to low language proficiency (Llácer et al., 2007). In our study, the data has national level coverage and uses objective sources, thus excluding recall and selection bias. Nevertheless, it is important to stress that contact with OPMH services only detects those who sought help there and not all young women experiencing mental disorders. We also lack information about those who used a private psychologist, psychiatrist or those who get inpatient treatment. However, inpatient services constitute only five percent of contacts with mental healthcare services (Norwegian Directorate of Health, 2019). According to Ngui et al. (2010) over two thirds of all people affected by mental disorders do not receive the care they need, mainly due to not contacting healthcare services. Thus, even possessing information about the whole population, we were unable to determine the real scope of the problem and the impact mental disorders may have on school completion.

Furthermore, our findings did not support the confounding hypothesis, as the association between use of OPMH services and school completion remained virtually unchanged when controlling for migrant background and parental education. This was unexpected, as previous studies found that several family background variables such as parental education and migrant background had an influence on both mental health and educational outcomes among young people (George, 2013; Reiss, 2013). Nonetheless, results from a Swedish register-based study found the effect of mental disorders on school completion was robust, despite adjustment for several family background variables (Brännlund et al., 2017), as in our study.

Our study aimed to fill the gap on the lack of research looking at differences between young women with migrant background in the association between use of OPMH services and school completion. As migrants and descendants make up a growing part of the society in many parts of the world, their mental health and its impact on other aspects of life is important to map. Based on previous research pinpointing migrants’ increased risk of development of mental disorders and lower school completion rates than the majority population, we hypothesized that the effect of use of OPMH services on school completion would be even stronger for migrants compared to descendants and the Norwegian majority. Additionally, we expected the effect to vary among migrants. The interaction analysis showed that the effect of use of OPMH services on school completion varied between migrants, descendants and the Norwegian majority. We, unexpectedly, found that descendant women had higher probability of completing school when using OPMH services compared to the Norwegian majority and migrant women from Nordic and Western countries. We also found use of OPMH services to have less adverse effect on school completion among migrant women from Eastern Europe, Asia and Africa. The strongest effect was found for majority women, migrant women from Nordic and Western countries and descendant women. This might be due to the greater social mobility and higher educational aspirations among descendants, and some migrant groups in the Norwegian society. A previous study suggested that descendants and several groups of migrants seem to have similar or higher educational attainment than the Norwegian majority when comparing groups with similar socioeconomic background (Hermansen, 2016). Furthermore, descendants, but also migrants, might have higher educational aspirations and expectations of life, as they report their parents having higher educational ambitions on their behalf compared with the Norwegian majority (Friberg, 2016). Also contrary to our expectations, the effects of OPMH service use on school completion was not stronger for migrants than for the Norwegian majority. While the above argumentation about educational aspirations may also apply, the unexpected findings could relate to help seeking. We know that migrants seek healthcare services to a lesser extent than the Norwegian majority (Abebe, Lien, & Elstad, 2017; Straiton et al., 2014, 2019). Additionally, we might expect migrants to experience greater barriers to seeking help for mental disorders than non-migrants. Thus, those who are in contact with OPMH services may be in a better position in terms of SES than those who do not seek help. This could mean that a larger proportion of resourceful young women with migrant background use OPMH services than resourceful majority women, resulting in the unexpected finding that descendants fare better and migrants do no worse regarding school completion, when using OPMH services than the majority population. However, it is important to emphasize that this does not mean descendants or migrants are not at a disadvantage having had a mental disorder.

### 4.1 Limitations

Our study has several other limitations. Firstly, the age period for the main exposure is measured when individuals are aged 16–19 years. This age period is slightly overlapping with the period of the outcome, as the majority of students complete upper-secondary education when aged between 19 and 21. Ideally, the exposure should be measured prior to enrolment in upper-secondary education. However, due to the limited number of years the registry data was available, and in order to use the same number of exposure years for each cohort while simultaneously maximising the use of the available information, we had to observe individuals when aged 16–19 years.

Secondly, we do not differentiate between different mental disorders in this study. Previous studies found that internalizing and externalizing disorders have a different impact on educational attainment. The effect of externalizing problems, such as conduct and attention problems, on educational attainment seems to be stronger than for internalizing problems such as depression and anxiety (e.g. Evensen et al., 2016; McLeod et al., 2012). However, research comparing those two categories is inconsistent.

A third limitation of this study is our operationalization of migrant background. To ensure the statistical power of our analysis and the anonymity of individuals included in our study, we were only able to divide migrants into four groups. However, we are aware of that the variety within the groups may influence our results and that these broad categories will mask important differences. More details about migrant background are needed to really understand how and why these groups may be different from each other.

Finally, we lack information on several other variables that could potentially confound or contribute to a better understanding of the association between use of OPMH services and school completion. Unmeasured factors such as one's own educational aspirations or parental expectations regarding one's educational attainment could contribute to understanding the underlying mechanisms of the association between mental disorder and school completion. For instance, Reisel, Hermansen, and Kindt (2019) suggested that research based on registry data could benefit from including survey-based data on ambitions, motivations and mechanisms that drive adolescents when it comes to educational choices and academic achievements. Other research also pinpointed such factors as possible confounders in the association between health and educational outcomes (Lynch & von Hippel, 2016). We agree and suggest that future research should incorporate personal perspectives as well as parental aspirations, since these could contribute to explaining the differences between majority, descendant and migrant women regarding OPMH service use and educational attainment. We also recommend future research to address the limitations of our study.

## 5 Conclusion

In summary, use of OPMH services is negatively associated with upper-secondary school completion among young women, and is robust to adjustments for potential confounding variables. Furthermore, our hypothesis that migrant women who use OPMH services would have a lower probability of completing upper-secondary education than their Norwegian and descendant counterparts was partly rejected. We found that young descendant women who use OPMH services had significantly higher probability of completing upper-secondary school compared to their Norwegian counterparts and migrant women from Nordic and Western countries. Migrant women from Eastern Europe, Asia and Africa did not significantly differ neither from the majority nor from descendants. However, this does not mean that having had a mental disorder is not a disadvantage for descendants. Migrants and descendants are less likely to use healthcare services than majority women, thus those who use OPMH services may be more resourceful than both migrants who do not seek help, as well as the majority in contact with OPMH services.

Our findings are of great importance as they show the negative impact of mental disorders on school completion in young women, which can subsequently influence other aspects of life and result in lower salary, limited workforce participation and have adverse effects on health. Such limitations have costs for the whole society, due to reduced ability to gain social capital, and increased dependency on welfare services of people affected by mental disorders.

## Financial disclosure statement

This study was funded by the Research Council of Norway through the ‘Women's Health programme’ project number 273262.

## Declaration of competing interest

There is no conflict of interest.
